# The normal width of the lateral incisors in the maxilla in an European population

**DOI:** 10.1007/s00784-025-06235-2

**Published:** 2025-02-26

**Authors:** Irene Artioli, Aline Ndayisaba, Jean-Pierre Ndayisaba, Adriano Giacomo Crismani

**Affiliations:** 1 Department of Dental and Oral Medicine and Cranio-Maxillofacial and Oral Surgery, Medical University of Innsbruck, MZA, Anichstr. 35, Innsbruck, A-6020 Austria; 2 University Hospital for Conservative Dentistry and Periodontology, Medical University of Innsbruck, Anichstraße 35, A-6020 Innsbruck, Austria; 3 Clinical Department of Neurology, Medical University of Innsbruck, Anichstraße 35, Innsbruck, A-6020 Austria

**Keywords:** Dental anomalies, Maxillary incisors, Width, Formula, Hypoplasia

## Abstract

**Objectives:**

Dental anomalies are clinical alterations that originate during the tooth formation process. This prospective study aims to establish the norm and hypoplasia values for maxillary lateral incisors. Additionally, it aims to describe the relationship between the width of the lateral and the central incisors, considering gender, age, and body height.

**Materials and methods:**

The first 103 consecutive patients meeting the inclusion criteria were included in the study. The upper jaw of each patient was scanned, and the scans were saved as standard tessellation language (STL) files. The upper incisors of each digital cast were measured on the computer. Two examiners performed the same measurements independently.

**Results:**

The average size of the maxillary central incisor was 8.59 ± 0.51 mm, while the average size of the maxillary lateral incisor was 6.64 ± 0.46 mm. A formula describing a relationship between the central (x) and lateral (y) incisors was defined with a linear regression analysis y = 1.88 + 0.55 * x. Width of maxillary incisors [11, 12, 21, 22] increased with height (*p* = 0.028, *p* = 0.016, *p* = 0.016, *p* = 0.046 respectively).

**Conclusions:**

Hypoplasia of the maxillary lateral incisor can be defined for teeth with a mesiodistal width less than 6.18 mm in a patient that is represented with the test group. A relationship between the maxillary lateral incisors and central incisors could have been established by means of a formula.

**Clinical relevance:**

The width of the maxillary lateral incisors allows a precise orthodontic and prosthetic planning.

## Introduction

Already Leonardo Da Vinci analyzed the anatomy of the teeth [1]. In 1870, Mühlreiter published the book “Anatomy of the Human Dentition”, which is still considered the basis of many current publications on the anatomy of teeth [2]. The maxillary incisors play the most important role in smile aesthetics [3].

Dental anomalies are clinical alterations which have their origin during the tooth formation process. This anomalies with various clinical manifestations and degrees of severity, from mild to severe cases are characterized by disturbances in number, size, shape, position and structure of the teeth [4]. Dental anomalies lead to aesthetic and functional problems. Genetic factors play an important role for the development of dental anomalies [5]. Radiographic examinations show that over 60% of children with anomalies in their primary dentition, have also anomalies in the permanent dentition [6]. These anomalies can be unilateral or bilateral, with a higher incidence on the left side [7]. A peg-shaped tooth for example is defined by certain criteria. If the incisal mesiodistal width of the crown is shorter than the cervical width then it gets classified as peg-shaped tooth [7, 8]. The general prevalence of peg-shaped maxillary lateral incisor is 1.8% and the prevalence of maxillary lateral incisor agenesis is 1–3% [9]. Women are affected 1.35 times more often than men [8]. It is therefore essential for dental treatment planning to take this into account in order to achieve an optimal aesthetic result [3].

Aesthetic dentistry tries to find geometric or mathematical rules that describe the proportion of the anterior teeth [10]. The ratio of the lateral-to-central maxillary incisors is essential to achieve an aesthetic and lovely smile. Unfortunately, there is little scientific data about these proportions [11]. The Golden Ratio 1.61: 1 that occurs in nature has already been used in architecture [12]. Lombardi was the first to propose the use of the “golden proportion” in dentistry [10]. The “golden proportion” was used to determine an ideal tooth size [3]. This ratio, especially the 62% width-length ratio of the maxillary central incisors is the least aesthetic [12]. So, this concept was rejected [13] because it was to be found only in 14–25% of the studied patients [10]. German et al. published a simplified method that describes the relation of the incisor widths in the mesio-distal direction [13]. This states that the width of the lateral incisor in the maxilla is equal to the width of the central incisor minus 2 mm [13].

There are published studies that have measured the width of the maxillary incisors [14–22]. Crown height and width are, in the available literature, very heterogeneously distributed between the different patient populations [18]. In these studies, Asian, African and American patient cohorts were analyzed geographically. These clinical differences have also been demonstrated from a genetic perspective. For example East Asian and Native Americans have larger teeth compared with Southeast Asians [23].

The treatment for patients with peg-shaped incisors is dependent on the patient’s expectation and the expertise of the clinician. The most drastic solution is the extraction of the teeth and a replacement with an implant [24]. It’s possible to make an multidisciplinary treatment plan for example with periodontal surgery, orthodontic movement of the teeth and prosthetic restorations [24, 25]. The orthodontic treatment has the aim to open the space mesial and distal to the peg-shaped tooth and to enable the restauration for a normal-sized lateral incisor [26].

Therefore, it is important to determine a definition of the tooth width of the incisors also for a European patient population. There are patients who don’t have a peg-shaped maxillary lateral incisor but still smaller maxillary lateral incisors. This prospective study aims to define the norm and hypoplasia values for the lateral incisors in the maxilla. In addition, we wanted to describe in which relationship the width of the lateral incisors should be compared to that of the central incisors, with consideration of gender, age and height.

## Methods

Approval of the study design was obtained by the Ethics Committee of the Medical University Innsbruck (Innsbruck, Austria; study ID: 1029/2022).

In this prospective analysis, the first consecutive 103 patients undergoing treatment at the Department of Dental and Oral Medicine and Cranio-Maxillofacial and Oral Surgery (Medical University of Innsbruck, Austria) between 4/2022 and 3/2023 and who satisfy the inclusion criteria were included. The inclusion criteria were age between 18 and 40 years, completely erupted incisors in the upper jaw and European (geographical) origin. The exclusion criteria were prosthetic restorations of the maxillary anterior teeth, missing central and/or lateral incisors, past approximal enamel reduction, and cleft lip.

**Fig. 1:**
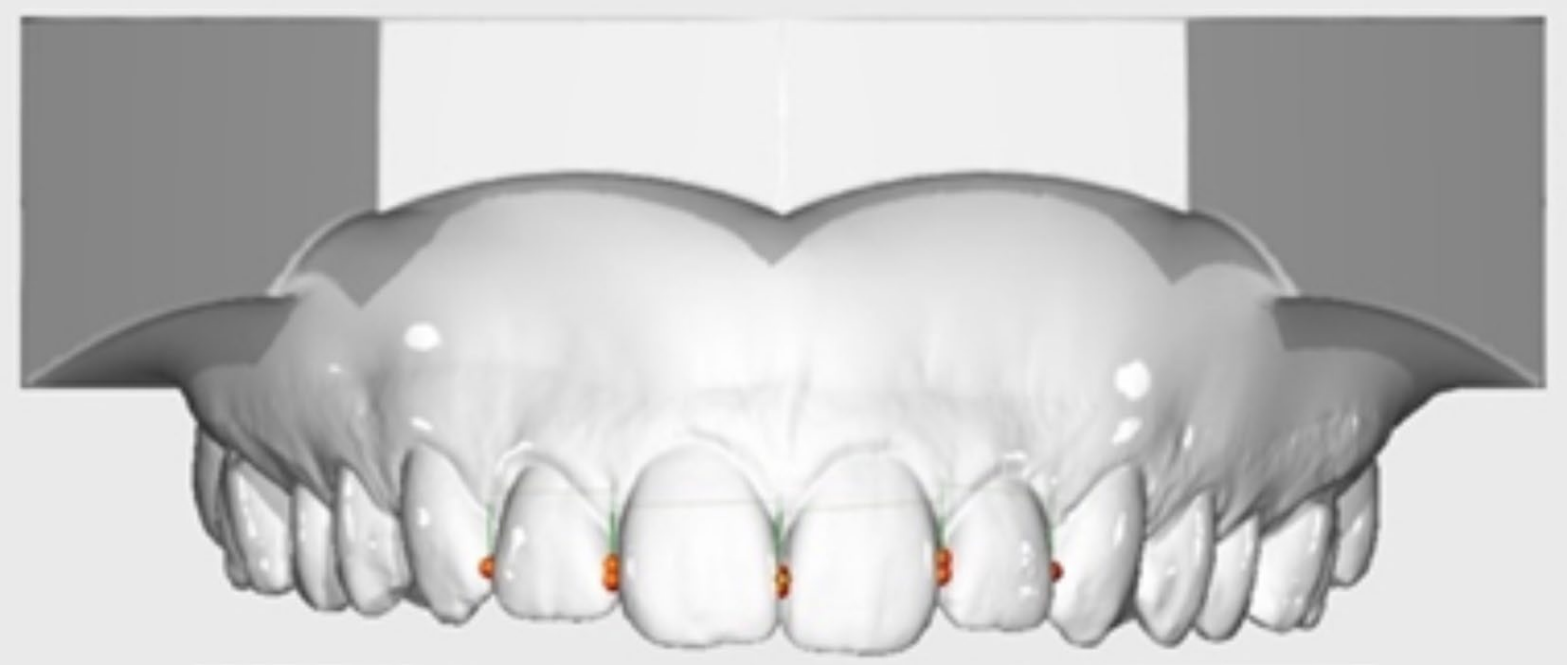
Measurements of the teeth on a digital model in OnyxCeph3™. The landmarks were placed at the distal and mesial contact points on the tooth crown (created with OnyxCeph3™). These landmarks are visible as red dots

103 patients were recruited for a single intraoral scan (iTero Element 2, version 1.12.0.990; Align Technology). Prior to that the participants signed a consent agreement in which they agreed to taking part in this study and the use of their data. The upper jaw of each patient was scanned, and the scans were saved as standard tessellation language (STL) files and uploaded to the internet for transformation into digital casts. The upper incisors of each digital casts were measured applying the OnyxCeph3™ Pro analysis software (version 3.2.147; Image Instruments, Chemnitz, Germany) on the computer. The software offers landmarks that can be placed at the points to be measured. In our case, they were placed at the distal and mesial contact points, taking care that they were placed on a line. Thus, the shortest mesiodistal distance of each upper incisor was measured. Two different examiners performed the same measurements independently. All measurements on the digital models were performed three times by both examiners under identical conditions with intervals of one month. Each tooth was measured a total of six times (Fig. 1).

In addition to the intraoral scans, all 103 patients were questioned about height, gender, and age.

An Excel table was created to record the data. All study-relevant data was recorded in this table in indirect person-related form. The table didn’t contain any data for the direct identification of the patients (such as name or date of birth) but contains a consecutive numbering without content-related reference.

Measurements were entered in a spreadsheet (Microsoft Excel, Redmond, WA, USA) and analyzed in SPSS Statistics (v. 29; IBM SPSSInc., Armonk, NY, USA). The six mesiodistal width values of each tooth were averaged to minimize errors and the mean value was used for statistical analysis. The Kolmogorov-Smirnov test was used to check for normal distribution. Descriptive statistics were expressed as mean values ± standard deviation unless otherwise stated. Counts (percentages) were used for categorical variables. Graphs such as histograms, box plots and scatter/dot plots were used to present the data. Differences between left and right incisors were analyzed using the paired-samples t-test. Group differences between men and women, and between age groups (categorized as ≤ 25 years and > 25 years) were analyzed using independent-samples t-test. Linear regression was used to analyze the relationships between incisor width and body height, and between lateral and central incisors. The significance level for all statistical tests was set at a p-value of < 0.05.

## Results

The data of all 103 probands were evaluated. The six mesiodistal width values of each tooth were put together for the calculation of the statistics.

A total of 51 women (49.5%) and 52 men (50.5%) participated at the study. 38 participants (36.9%) were ≤ 25 years old and 65 (63.1%) were > 25 years old. The average age of the participants was 26.1 ± 2.9 years, and the average height was 173.9 ± 9.6 cm. The average age of the women was 25.9 ± 2.8 years, and the average height was 166.9 ± 6.0 cm. The average age of the men was 26.2 ± 3.0 years, and the average height was 180.8 ± 7.2 cm (Table 1).

**Table 1:** Description of the participants divided by gender. For each gender, the percentage of participants, mean height and mean age were provided

	Women (*n* = 51)	Men (*n* = 52)
Gender (%)	49.5	50.5
Mean body height (cm)	166.9 ± 6.0	180.8 ± 7.2
Minimun	153.0	164.0
Maximum	180.0	196.0
Mean age (y)	25.9 ± 2.8	26.2 ± 3.0
Minimum	21.60	19.23
Maximum	35.6	33.82

### The size of the maxillary incisors

The mesiodistal average size of tooth 12 was 6.65 ± 0.48 mm, that of tooth 11 8.61 ± 0.51 mm, that of tooth 21 was 8.57 ± 0.52 mm and that of tooth 22 was 6.63 ± 0.47 mm.

It was found that the average size of all the central incisors of our patient population was 8.59 ± 0.51 mm and the average size of the lateral incisors was 6.64 ± 0.46 mm.

All measurements of both the examiners were averaged. Two way mixed Intraclass Correlation Coefficients using a consistency definition (95% confidence interval) were calculated: tooth 12 0.950 (CI 0.927–0.966), tooth 22 0.955 (CI 0.933–0.969), tooth 11 0.964 (CI 0.946–0.975) and tooth 21 0.967 (CI 0.951–0.978). The inter-rater variability therefore was excellent.

More details are provided in Table 2.

**Table 2:** The mean value of the mesiodistal width of teeth 12, 22, 11 and 21. The table also describes the mean values of the lateral and central incisors without distinguishing between right and left

	Mean (mm)	SD (mm)	95% CI for Mean (mm)
Tooth 12	6.65	0.48	6.56–6.75
Tooth 22	6.63	0.47	6.54–6.72
Tooth 11	8.61	0.51	8.51–8.71
Tooth 21	8.57	0.52	8.47–8.67
Centrals	8.59	0.51	8.49–8.69
Laterals	6.64	0.46	6.55–6.73

SD standard deviation, CI confidence interval

### The relationship between the lateral and the central maxillary incisors

The linear relationship summarizing the correlation between the central and lateral incisors can be obtained by regression analysis:

$$\:y=1.88+0.55*x$$

**Fig. 2:**
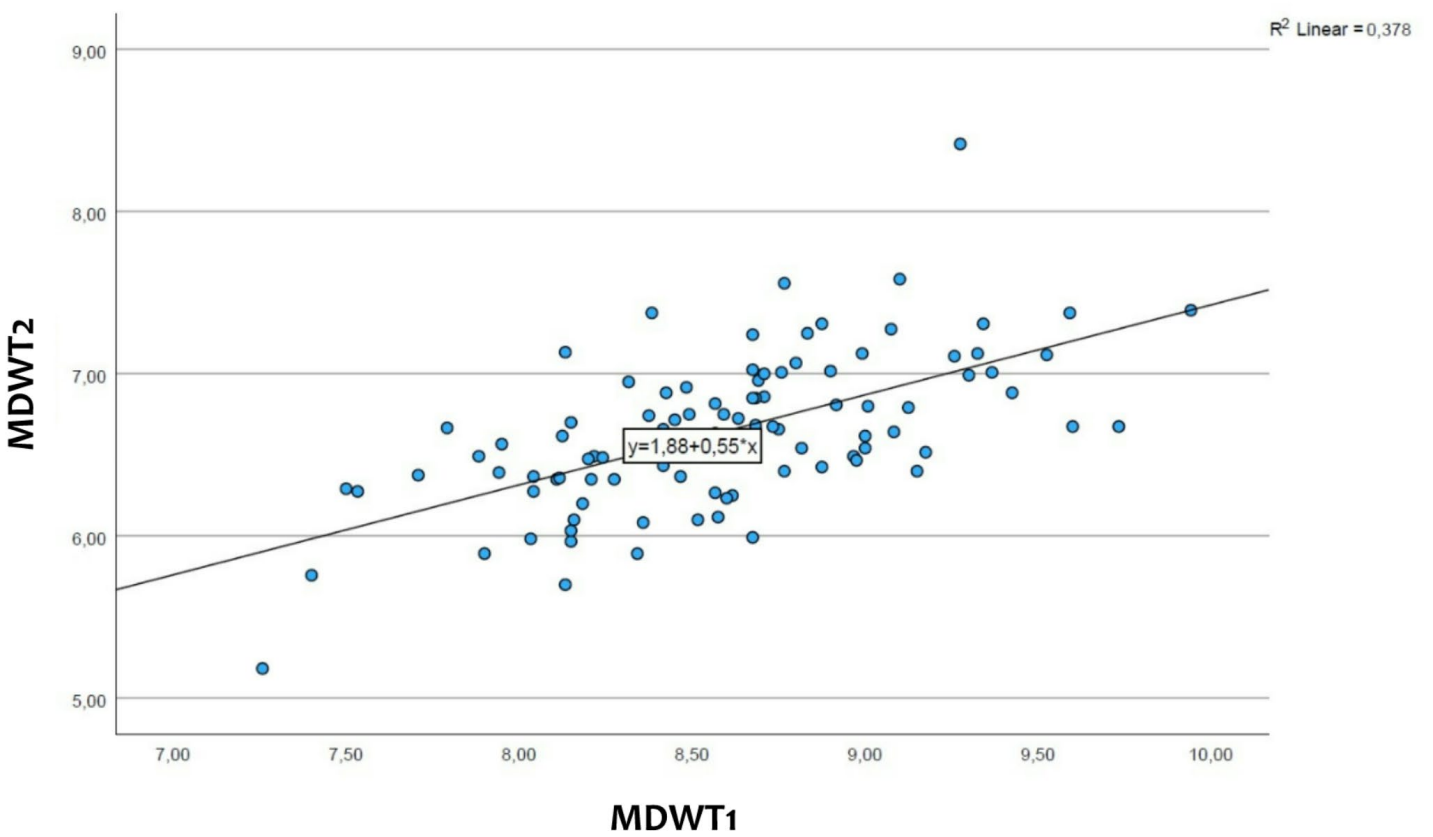
The relationship between the central (MDWT1) and the lateral incisors (MDWT2) (created with SPSS). On the x-axis is represented the mesiodistal width of all the central incisors, while on the y-axis that of the lateral incisors. From the relationship between the two sizes results a straight line

Y represents the mesiodistal width of the maxillary lateral incisor, while x represents the mesiodistal width of the maxillary central incisor. The formula also includes a constant of 1.88 (*p* = 0.003) and a regression coefficient of 0.55 (*p* < 0.001) (see also Fig. 2).

### The gender differences for the maxillary incisor width

**Fig. 3:**
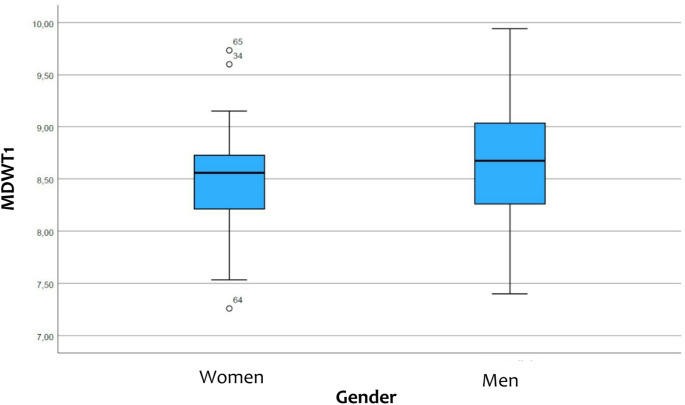
The box-plot shows the comparison of the width of the central maxillary incisor (MDWT1) in men and women (created with SPSS)

**Fig. 4:**
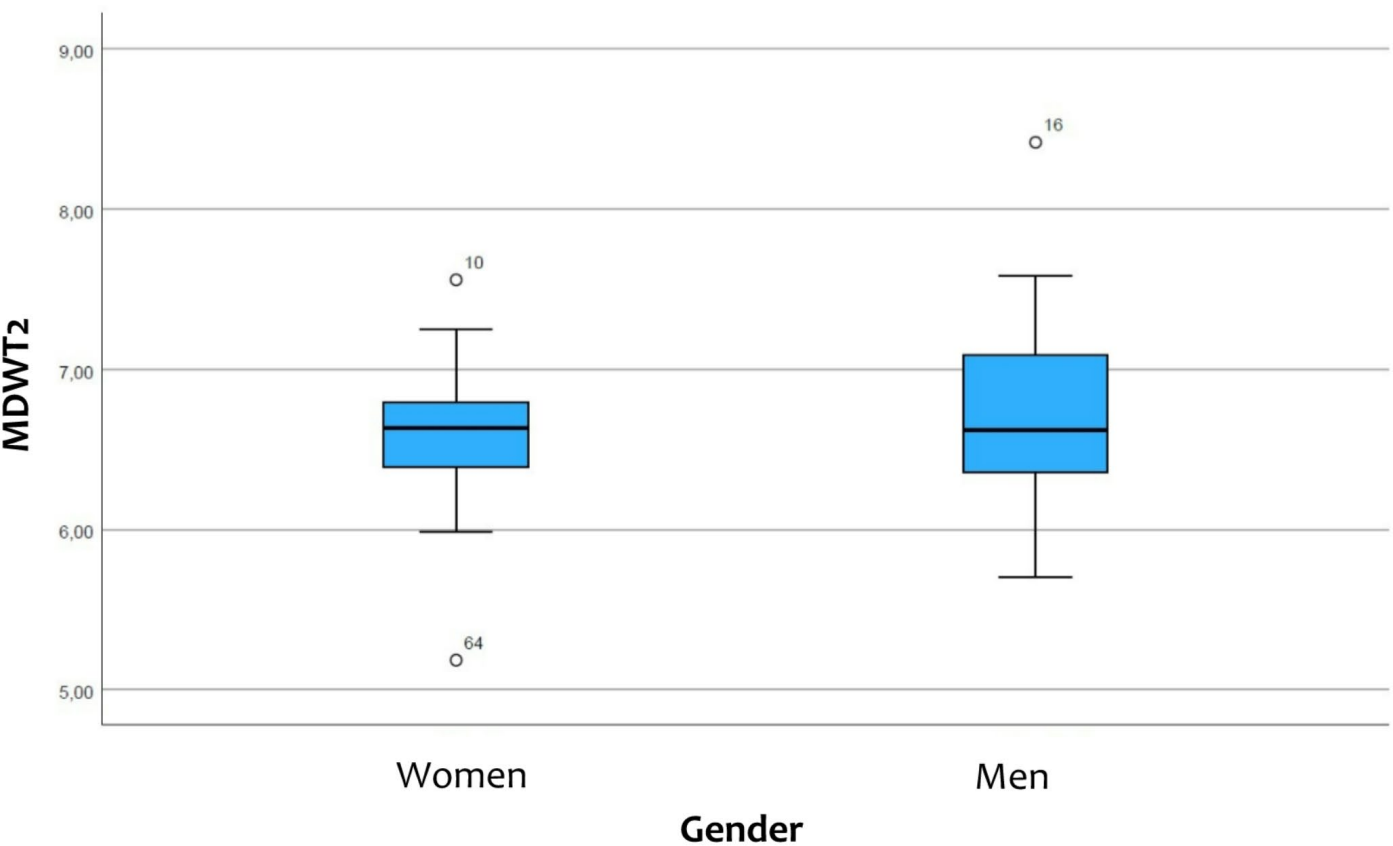
The box-plot shows the comparison of the width of the maxillary lateral incisor (MDWT2) in men and women (created with SPSS)

For women was the average width of tooth 12 6.61 ± 0.40 mm, that of tooth 11 was 8.53 ± 0.46 mm, that of tooth 21 was 8.50 ± 0.46 mm and that of tooth 22 was 6.58 ± 0.40 mm. The general size of the central maxillary incisor in women was 8.51 ± 0.45 mm and that of the maxillary lateral incisor was 6.59 ± 0.37 mm (Figs. 3 and 4).

For men the average width of tooth 12 was 6.70 ± 0.54 mm, that of tooth 11 was 8.69 ± 0.55 mm, that of tooth 21 was 8.64 ± 0.56 mm and that of tooth 22 was 6.68 ± 0.54 mm. The general size of the central maxillary incisor in men was 8.66 ± 0.55 mm and that of the maxillary lateral incisor was 6.70 ± 0.53 mm (Figs. 3 and 4).

No statistically significant difference was found between the maxillary incisors in men and women (Table 3).

**Table 3:** The mesiodistal width of the maxillary incisors divided by gender. The table also shows the mean values of the lateral and central incisors without distinction between the right and the left side

	Women			Men			
	Mean (mm)	SD (mm)	95% CI of Mean (mm)	Mean (mm)	SD (mm)	95% CI of Mean (mm)	Paired t-test (*p*)
Tooth 12	6.61	0.40	6.49–6.72	6.70	0.54	6.55–6.85	0.343
Tooth 22	6.58	0.40	6.47–6.69	6.68	0.54	6.53–6.83	0.267
Tooth 11	8.53	0.46	8.40–8.66	8.69	0.55	8.54–8.84	0.112
Tooth 21	8.50	0.46	8.37–8.63	8.64	0.56	8.49–8.80	0.162
Centrals	8.51	0.45	8.39–8.64	8.66	0.55	8.51–8.82	
Laterals	6.59	0.37	6.49–6.70	6.70	0.53	6.54–6.84	

SD standard deviation, CI confidence interval

*Difference statistically significant at *p* < 0.05

### Differences for the maxillary incisor width regarding age

**Fig. 5:**
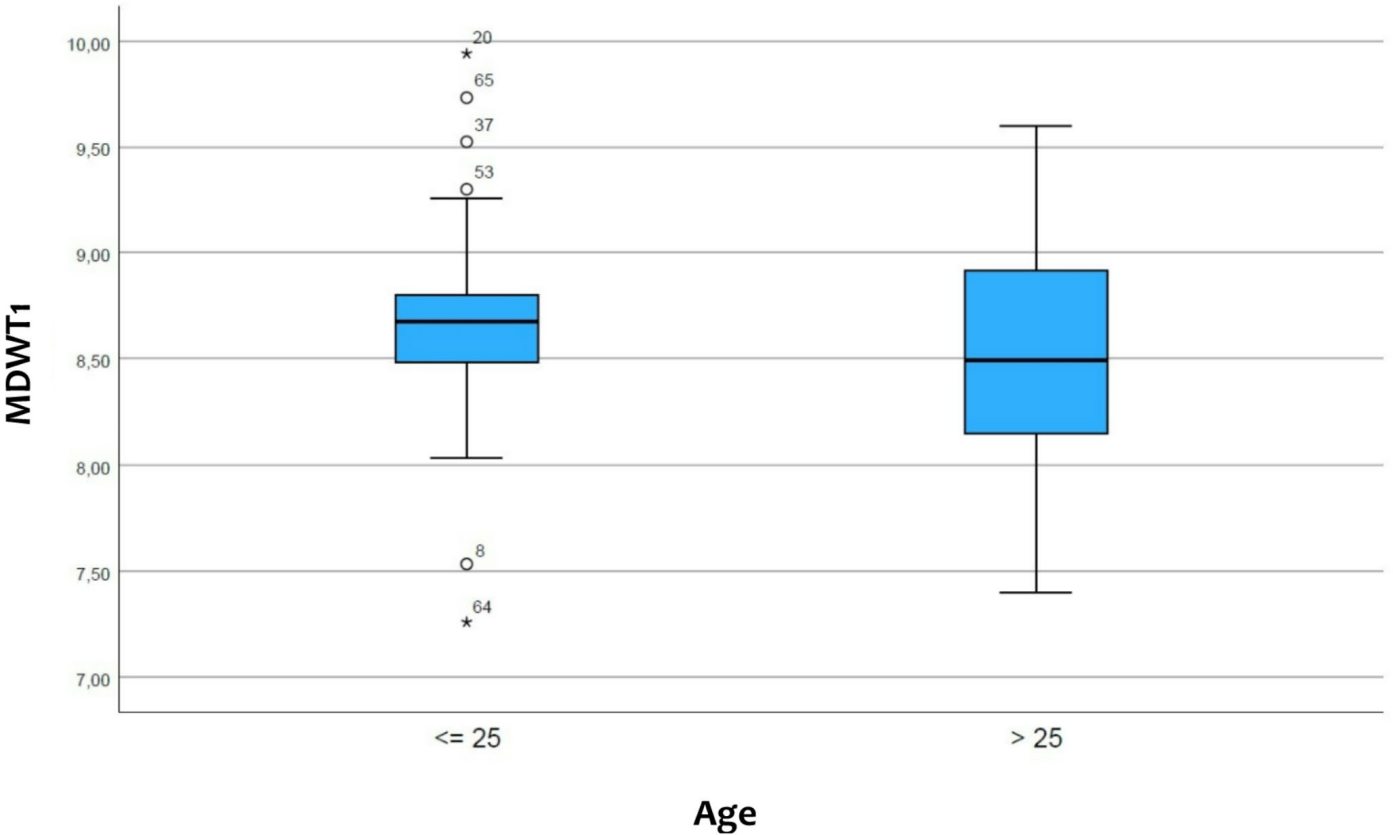
The box-plot shows the comparison of the width of the maxillary central incisor (MDWT1) in participants younger or older than 25 years (created with SPSS)

**Fig. 6:**
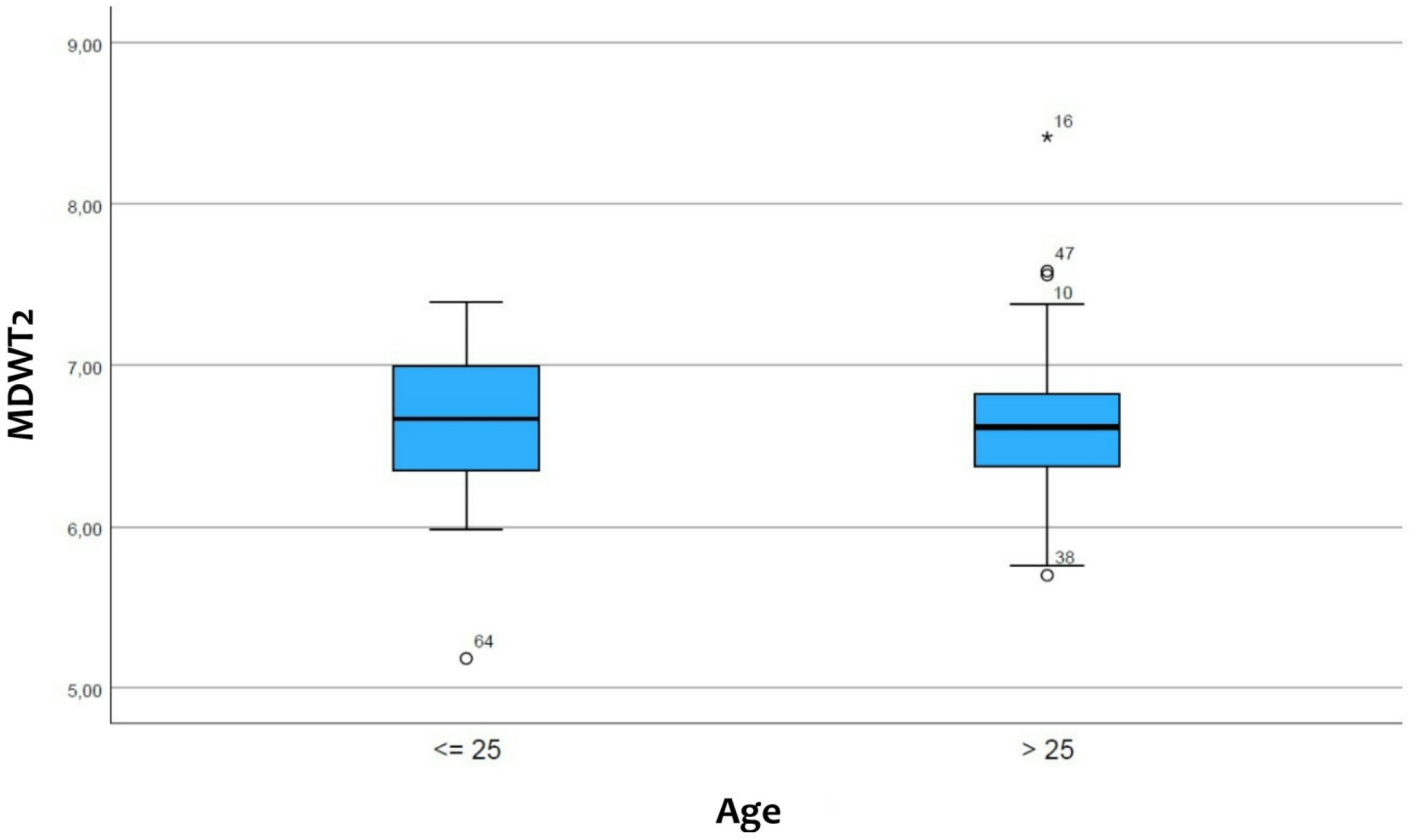
The box-plot shows the comparison of the width of the maxillary lateral incisor (MDWT2) in participants younger or older than 25 years (created with SPSS)

The average tooth width for the test subjects < 25 years old is as follows for tooth 12 6.66 ± 0.46 mm, as for tooth 11 8.67 ± 0.53 mm, as for tooth 21 8.67 ± 0.50 mm and as for tooth 22 6.63 ± 0.46 mm. The general size of the maxillary central incisor in younger probands was 8.67 ± 0.50 mm and that of the maxillary lateral incisor was 6.65 ± 0.45 mm (Figs. 5 and 6).

For probands aged > 25 years the average width of tooth 12 was 6.65 ± 0.49 mm, that of tooth 11 was 8.57 ± 0.50 mm, that of tooth 21 was 8.51 ± 0.53 mm and that of tooth 22 was 6.63 ± 0.49 mm. The general size of the maxillary central incisor in older probands was 8.54 ± 0.51 mm and that of the maxillary lateral incisor was 6.64 ± 0.46 mm (Figs. 5 and 6).

No statistically significant differences were found between the participants older or younger than 25 years (Table 4).

**Table 4:** The mesiodistal width of the maxillary incisors are divided by the age of the participants older or younger than 25 years. The table also shows the mean values of the lateral and central incisors without distinction between the right and the left side

	≤ 25 years			> 25 years			
	Mean (mm)	SD (mm)	CI (mm)	Mean (mm)	SD (mm)	CI (mm)	Paired t-test (*p*)
Tooth 12	6.66	0.46	6.51–6.82	6.65	0.49	6.53–6.77	0.876
Tooth 22	6.63	0.46	6.48–6.78	6.63	0.49	6.51–6.75	0.954
Tooth 11	8.67	0.53	8.50–8.84	8.57	0.50	8.45–8.70	0.355
Tooth 21	8.67	0.50	8.51–8.83	8.51	0.53	8.38–8.65	0.149
Centrals	8.67	0.50	8.50–8.83	8.54	0.51	8.42–8.67	
Laterals	6.65	0.45	6.50–6.79	6.64	0.46	6.53–6.76	

SD standard deviation, CI confidence interval

*Difference statistically significant at *p* < 0.05

### The body height differences for the maxillary incisor width

**Fig. 7:**
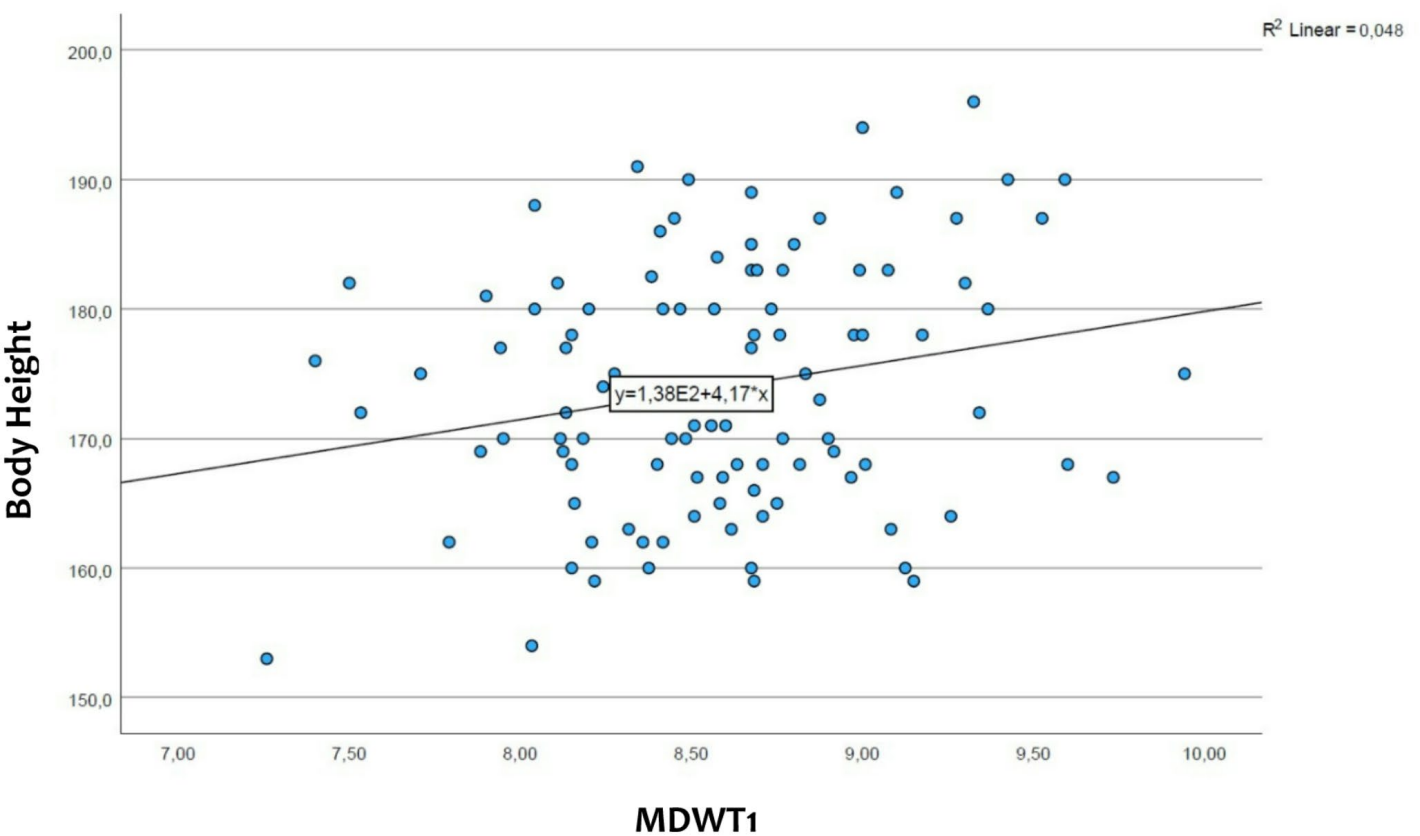
Comparison of the width of the maxillary central incisor (MDWT1) and the body height of the participants (created with SPSS). On the x-axis is represented the mesiodistal width of all the central incisors, while on the y-axis the body height of the participants. From the relationship between the two sizes results a straight line

**Fig. 8:**
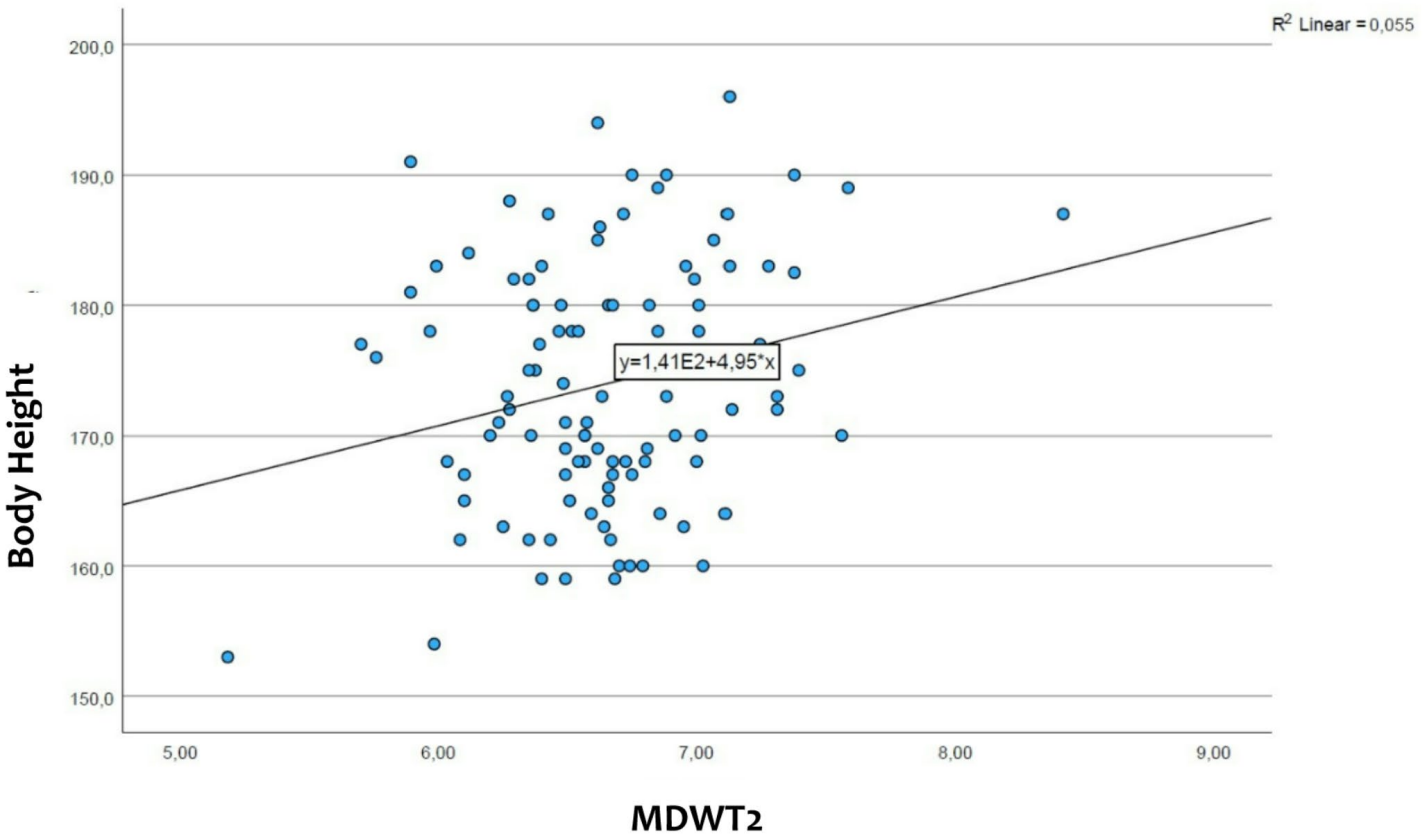
Comparison of the width of the maxillary lateral incisor (MDWT2) and the body height of the participants. On the x-axis is represented the mesiodistal width of all the lateral incisors, while on the y-axis the body height of the participants. From the relationship between the two sizes results a straight line

Regression analysis revealed a correlation between the size of maxillary incisors and body size, i.e. greater height is associated with larger teeth. Statistically significant p-values were found for all the maxillary incisors, with a p-value of 0.028 for tooth 12, a p-value of 0.016 for tooth 22, a p-value of 0.016 for tooth 11, and a p-value of 0.046 for tooth 21. (see also Figs. 7 and 8)

## Discussion

### The size of the lateral and central maxillary incisors

Until now it was not possible to accurately determine and calculate the mesiodistal width of the upper incisors for a European population, and therefore indicate at what value there is hypoplasia of the lateral incisor of the upper jaw. In our study we found that the mean value of the right central incisor was 8.61 ± 0.51 mm, that of the right lateral incisor was 6.65 ± 0.48 mm, that of the left central incisor was 8.57 ± 0.52 mm and that of the left lateral incisor was 6.63 ± 0.47 mm. In a study on a Nepalese population it was found that the mean value of the right central incisor was 8.62 ± 0.62 mm, that of the right lateral incisor was 6.97 ± 0.74 mm, that of the left central incisor was 8.65 ± 0.55 mm and that of the left lateral incisor was 7.11 ± 0.78 mm [14]. In a study on a Kurdish population it was found that the mean value of the right central incisor was 8.48 ± 0.51 mm, that of the right lateral incisor was 6.38 ± 0.49 mm, that of the left central incisor was 8.44 ± 0.43 mm and that of the left lateral incisor was 6.40 ± 0.55 mm [19]. These results indicate a difference in the width of the upper incisor in the different ethnic groups.

With our study we could define a hypoplasia of the maxillary lateral incisor for teeth with a mesiodistal width smaller than 6.18 mm. This result is important so that the clinician can easily understand whether a patient needs an enlargement of the lateral incisor or not. This makes it possible to establish a clear diagnosis for the patient and thus to prepare, if necessary, the spaces with an orthodontic treatment prior the construction of this tooth.

After making an accurate diagnosis, it is also important to be able to calculate precisely how wide the upper lateral incisor should be, taking into consideration the width of the maxillary central incisors. This was made possible by the following formula:

$$\:y=1.88+0.55*x$$

As previously described, indicates the variable y the mesiodistal width of the maxillary lateral incisor and the variable x indicates the mesiodistal width of the maxillary central incisor. In the study of German et al. the authors found a method to calculate the width of the upper lateral incisor, in which they subtract 2 mm from the width of the upper central incisor without taking geographical ethnic differences into account. The formula that we found in our study is only accurate for the European population.

### The relationship between the size of the maxillary incisors and the body height

In previous studies, the facial dimensions of three ethnicities were compared with the width of the maxillary incisors. The authors analyzed individuals of Asian, African-American and white ethnicities and they didn’t find a correlation between the facial dimensions and the mesiodistal width of the maxillary anterior teeth except in Asian women [17].

In the literature we could find a positive correlation between permanent tooth size and final body height [27]. In our study we analyzed the relationship between the maxillary incisors size and the body height, and we also found a positive correlation between the width of the maxillary incisors and the body height (statistically significant).

### The relationship between the size of the maxillary incisors and the gender

Male probands have larger teeth than female probands, because of the more effective promoting effect of the Y chromosome on tooth growth than that of the X chromosome [27].

In our study we found also that men have bigger teeth than women, but the difference is not statistically significant.

### The relationship between the size of the maxillary incisors and the age

In previous studies it was found that the thickness of the tooth depends on the chronological age. The enamel thickness begins to decrease at an age of 50 [28]. In our study we observed a population younger and older than 25 and we found that the younger population had slightly bigger teeth than the older. These differences were not statistically significant.

### Limitations

The number of probands was limited to 103 patients to give a mean value of the maxillary incisors as accurate and representative as possible. In the case of epidemiological studies, the more patients are included, the more precise are the results. For future projects, it might be possible to analyze a larger number of patients.

Due to the different sizes of the incisors in relation to the geographical origin of the subjects, it is recommended to use the formula found only for a European patient population. For future projects, it might be possible to find a new formula for other patient populations.

### Clinical relevance

Thanks to the formula found in this study, it is possible to determine the precise size of the maxillary lateral incisors by knowing the width of the central ones. This allows for more precise planning and interdisciplinary collaboration. The orthodontist knows how much to open the mesial and distal spaces of the upper lateral incisors, and the prosthodontist knows how much the reconstruction of the tooth needs to be widened.

The knowledge of the ideal tooth size allows therefore excellent results from an aesthetic and functional perspective.

## Conclusions

Within the limitations of this study, the following conclusions for an European population were drawn:

1. a hypoplasia of the maxillary lateral incisor can be defined for teeth with a mesiodistal width smaller than 6.18 mm;
2. it is possible to calculate the mesiodistal width of the maxillary lateral incisor with the following formula:

$$\:y=1.88+0.55*x$$

3. men have bigger teeth than women.
4. The bigger the body height, the bigger the teeth.

## Data Availability

No datasets were generated or analysed during the current study.
